# AI integration in healthcare simulation debriefing: support or substitute?

**DOI:** 10.1186/s41077-026-00448-5

**Published:** 2026-06-10

**Authors:** Lucy Stocks, Marc Place, Victoria Brazil, Ranjev Kainth, Belinda Lowe, Ibraaheem Khan, Prashant Kumar

**Affiliations:** 1 Department of Medical Education, NHS Lanarkshire, Bothwell, Scotland, UK; 2 School of Medicine, Dentistry & Nursing, University of Glasgow, Glasgow, Scotland, UK; 3 Department of Anaesthesia, NHS Forth Valley, Larber, Scotland, UK; 4 Faculty of Health Sciences and Medicine, Bond University, Gold Coast, Australia; 5 Faculty of Life Sciences and Medicine, King’s College London, London, UK; 6 Gold Coast Hospital Health Service, Gold Coast, Australia; 7 Department of Anaesthesia, NHS Greater Glasgow & Clyde, Glasgow, Scotland, UK; 8 School of Medicine, University of Dundee, Dundee, Scotland, UK

**Keywords:** Simulation, Debriefing, AI, Artificial intelligence, Large language models, LLMs, Generative AI, Reflection, Learning

## Abstract

Debriefing is widely recognised as a central mechanism for learning within healthcare simulation, enabling learners to reflect on clinical actions, decision-making, and team interactions. However, high-quality debriefing is resource-intensive, dependent on facilitator expertise, and increasingly challenged by the growing complexity and volume of data generated during modern simulation activities. Artificial intelligence (AI) offers emerging opportunities to augment aspects of debriefing by analysing performance data, structuring reflective dialogue, and supporting learning environments. This article explores the emerging role of AI within debriefing. Drawing on the current literature, we describe four modes of AI being integrated into debriefing practice: metric-based AI tutors, large language model-assisted debriefing tools, conversational chatbot debriefers and hybrid integrated AI systems. For each mode, we examine their underlying mechanisms, current applications, and current contributions to, and limitations within, debriefing. Using these four modes as a scaffold, we offer practical guidance for simulation practitioners considering the integration of AI tools within their own practice, including considerations related to faculty AI literacy, educational alignment, governance, and implementation. While the empirical evidence base is evolving, AI-driven approaches offer new ways of supporting facilitators in augmenting reflective practice. When implemented thoughtfully and with appropriate human oversight, the integration of AI into debriefing portends a new era supporting reflective learning within healthcare simulation.

**Supplementary Information:**

The online version contains supplementary material available at 10.1186/s41077-026-00448-5.

## Introduction

Debriefing, a facilitated conversation encompassing elements of performance analysis, feedback and critical self-reflection, is widely recognised as a crucial element of healthcare simulation [1–3]. Through facilitated reflective dialogue, learners typically examine their actions, decision-making, and team dynamics: a sociological learning process that enables the translation of lessons learnt into clinical practice [1–4]. Despite their critical role, debriefings can fail to realise their potential. Variability in quality and facilitator expertise, limited scalability, increasing faculty workload, and the difficulty of synthesising complex team-based performance data, all strain traditional debriefing approaches. Furthermore, simulation scenarios are increasingly a source of multimodal data, such as video recordings, performance metrics, physiological parameters, and communication transcripts [5], yet much of this information remains underutilised within the subsequent debriefing conversations.

Concurrently, the use of artificial intelligence (AI) is growing exponentially, reshaping the design and execution of multiple domains of healthcare simulation, including scenario design, delivery, assessment and virtual patient interaction [6–15]. These technologies introduce the possibility of augmenting how reflection is structured, how feedback is generated and delivered, and how performance data are interpreted. As such, they may address some of the inherent debriefing challenges that facilitators and learners encounter during debriefing and offer new avenues to optimise the learning environment.

However, our understanding of this evolving technology, its benefits and challenges, its risks and limitations, and its contextual nuances, remains in its infancy. As Cheng and McGregor [16] note, the uptake of AI within healthcare simulation has frequently been opportunistic, characterised by isolated demonstrations of feasibility rather than systematic integration. In response, they propose a structured ‘model of thinking’ to guide the implementation, evaluation, and adoption of AI. By situating AI-driven debriefing explicitly within this model, we aim to move beyond ad-hoc implementation of this emerging technology, toward a more conceptually grounded understanding of how AI may support reflective practice, while remaining attentive to the foundational elements required for responsible use.

In this article, we contextualise AI-driven debriefing within this emerging conceptual landscape. We describe four distinct modes of AI currently being integrated into debriefing practice, offering a structured framework for understanding and researching how different technological architectures interface with facilitators, learners, and performance data. For each mode, we examine the underlying mechanism, current applications, and associated limitations. We provide case vignettes to illustrate potential real-world applications of these technologies and offer pragmatic guidance for simulation practitioners on the responsible integration of these technologies, inviting consideration of whether they should function as a supportive adjunct or a potential substitute for faculty.

### Modes of AI used in debriefing

To provide a conceptual scaffold, we describe four distinct modes of AI currently being integrated into debriefing practice: metric-based AI tutors, LLM-assisted debriefing, conversational chatbot debriefers, and hybrid and integrated AI debriefing systems. These modes are differentiated not simply by the underlying technology, but by their functional architecture and pedagogical configuration; specifically, how AI interfaces with performance data, facilitators, and learners. Each mode supports debriefing in varying ways. For example, chatbot debriefers may use directed feedback to encourage reflection while LLM-assisted debriefs may adopt facilitated discussions. Whilst these modes are not mutually exclusive, they offer a structured framework for examining the mechanisms, contributions, and limitations of AI-driven debriefing. Please see Table 1 below which provides a summary of the key points of each of the four modes we have described. 

**Table 1:** Modes of AI-driven debriefing in healthcare simulation summary

Mode	Primary AI Configuration	Contribution to Debriefing	Key Limitations	Governance Considerations	Typical Use Context
Metric-based AI tutors	Rule-based algorithms, machine learning classifiers, simulator telemetry; quantitative benchmarking against predefined performance metrics	Standardised, objective feedback on technical performance; supports deliberate practice and scalable formative assessment	Limited to measurable parameters; less suited to behavioural or relational skills; may oversimplify complex performance	Transparency of scoring algorithms; validation of performance metrics; alignment with competency frameworks; interpretability of feedback	Procedural and technical skills training; skills labs; competency-based curricula
LLM-assisted debriefing	LLM analysis of transcripts, audiovisual logs, or multimodal analytics; facilitator- or learner-mediated outputs	Organises complex scenario data; enhances constructive alignment with ILOs; scaffolds facilitators; supports structured reflection	Output sensitive to prompt design; risk of fabrication or overgeneralisation; may increase cognitive load initially	Data privacy in transcript handling; AI literacy for users; human oversight of generated outputs; cybersecurity	Team-based simulations; complex scenarios; faculty development contexts
Conversational chatbot debriefers	Learner-facing LLM dialogue agents using structured prompts or scripts	On-demand, scalable reflective dialogue; repeatable communication skills practice; asynchronous access	Risk of sycophancy or insufficient critical challenge; limited non-verbal and relational sensitivity; vulnerability to bias	Reduced human oversight; informed consent; transparency of feedback generation; safeguarding psychological safety	Communication training; early clinical reasoning; self-led debriefing
Hybrid and integrated AI debriefing systems	Multi-component architectures combining rule-based logic, RAG grounding, multimodal analytics, and LLM synthesis	Greater traceability and architectural robustness; longitudinal tracking; integration of behavioural and performance data; potential redesign of reflective infrastructure	Technical complexity; high development cost; limited large-scale validation; system dependency	Algorithmic transparency; validation of integrated pipelines; governance embedded in design; curriculum alignment	Advanced simulation programmes; high-volume systems; algorithmic simulations

#### 1) Metric-based AI tutors

##### Definition and mechanism

Metric-based AI tutors are structured educational systems that utilise simulator telemetry and performance metrics to generate automated, individualised feedback following simulation-based tasks. These systems are most commonly deployed within procedural or technical skills training, where learner performance can be captured through quantifiable parameters such as instrument movement, time efficiency, error rates, or adherence to predefined algorithms or standards of care and performance. In contrast to LLM-driven systems, metric-based AI tutors rely primarily on rule-based algorithms, machine learning classifiers, or performance benchmarking models to evaluate competency against established criteria, with emphasis on quantifiable performance measurement rather than conversational reflection [17, 18].

##### Current applications

The development of AI-enabled metric tutors has been most prominent in procedural simulation, with several studies documenting their use. Mirchi et al. [17] developed a virtual operative assistant (VOA), an AI-driven system that uses machine learning to analyse instrument motion and procedural metrics in virtual reality simulation. The VOA provides structured, staged feedback, initially targeting foundational safety metrics, then progressing to more complex elements of the skill, such as instrument handling. A recent randomised control trial compared the VOA with remote expert instruction, reporting that learners receiving AI tutoring demonstrated improved technical performance and skill transfer [18]. Measures of cognitive load and emotional response were comparable between groups.

##### Contribution to debriefing

Metric-based AI tutors address several persistent challenges in debriefing of procedural skills. First, they provide highly quantifiable feedback on motor performance and safety parameters that may be difficult for human facilitators to observe in real time, particularly in group settings. Second, they standardise feedback delivery, reducing inter-facilitator variability, which could be integrated into competency-based curriculums. Third, they enable repeated deliberate practice with immediate formative feedback at a time convenient for the learner, thereby supporting self-led debriefing practice [19, 20].

**Table:** Case Vignette 1: An example of how a metric-based AI tutor can be used to allow for longitudinal development of surgical skills

Case Vignette 1: Metric-based AI tutors	
An obstetrics and gynaecology trainee practices laparoscopic salpingectomy using a virtual reality simulator. During each session, the AI system provides real-time feedback on instrument movement, efficiency, and error rates. At the end of the session, the trainee receives a summary comparing their performance against predefined metrics, with suggestions for targeted improvement. Performance is tracked across a 10-session programme, enabling both the trainee and supervisor to monitor progression. This AI system utilises machine learning to classify trainee performance and generative AI to provide a personalised and an actionable feedback report, which can then be used for critical self-reflection. The supervisor uses these data to identify learners requiring additional support, while progression decisions are informed by achievement of predefined competency thresholds.	

##### Limitations

Despite these advantages, metric-based AI tutors are inherently constrained by the nature of the data they can capture. Their utility is strongest in simulation activities where performance can be operationalised into measurable parameters. They are less suited to debriefing domains centred on interpersonal and behavioural skills, where nuanced interpretation and relational sensitivity are required. Additionally, while algorithmic evaluation may enhance standardisation, it raises governance considerations related to transparency of scoring systems, validation of performance metrics, and alignment with competency frameworks [21, 22]. Responsible implementation therefore mandates that performance benchmarks are evidence-based, interpretable, and integrated coherently into curricular structures in a transparent manner. At present, evidence remains concentrated within technical skills training, and further work is required to determine how these systems interface with broader reflective debriefing processes. Nevertheless, metric-based AI tutors currently represent one of the most empirically supported modes of AI-driven feedback in healthcare simulation.

#### 2) LLM-assisted debriefing

##### Definition and mechanism

LLM-assisted debriefing refers to the use of LLMs to analyse data generated from simulated scenarios or tasks, such as transcripts derived from audiovisual recordings or chat logs, and produce outputs intended to support human-led debriefings. These outputs may include structured summaries of events, suggested debriefing agendas, examples of specific questioning methods, or thematic mappings aligned to intended learning outcomes (ILOs). In this mode, the facilitator(s), or the learner(s) themselves in self-led debriefing contexts, retain interpretative authority, exercising judgement over what AI-generated information is incorporated, actioned, or discarded. The AI therefore functions as an analytic support tool rather than an autonomous debriefer. Its primary function is to assist facilitators, or learners themselves, in organising complex scenario data into focal points that can be deconstructed, analysed and reflected upon.

##### Current applications

Studies have piloted the use of general-purpose LLMs (via ChatGPT 4.0) to generate structured debriefing support materials from simulation transcripts [23, 24]. Facilitators in both studies reported valuing the structure provided by AI-generated summaries and suggested debriefing questions. Hong et al. [23] found that LLM outputs were perceived as helpful in targeting ILOs, although facilitators reported moderate cognitive load associated with integrating the technology into practice. Tscholl et al. [24] demonstrated that constraining model output through structured prompts, such as mapping behaviours to the Team-FIRST framework, improved usability and alignment with educational objectives. More technically sophisticated systems have extended beyond transcript-only approaches. Echeveria et al. [25] developed TeamVision, a multimodal learning analytics platform that captures scenario data and generates a facilitator-facing dashboard to guide the debriefing discussion. While most participants reported improved debrief structure and targeted discussion, some noted discrepancies between AI-generated outputs and their own observations. Benfatah et al. [26] integrated motion and speech analytics (via OpenPose and Deepgram) with LLM interaction.

##### Contribution to debriefing

LLM-assisted debriefing addresses several recognised challenges in current debriefing practice. First, it supports the organisation of complex, high-volume data, particularly in team-based immersive simulations, into coherent narrative summaries and discussion points, offering a degree of meaningful analytic augmentation. Second, it may enhance constructive alignment by explicitly linking observed behaviours to predefined ILOs or frameworks. Third, by generating questions or discussion themes, it may reduce the initial cognitive burden associated with synthesising events in real time. Importantly, this mode does not displace facilitator, or learner, judgement, but redistributes analytic labour. The human remains responsible for contextual interpretation, emotional containment, and pedagogical adaptation. In this sense, LLM assistance may function as a scaffold, particularly for novice faculty, whilst also supporting greater structural consistency across programmes, a theme echoed in broader reviews of generative AI in healthcare simulation [27–29].

In addition, LLM-assisted analysis may have potential applications within simulation faculty development. For example, structured transcript analysis or AI-generated summaries of debriefing interactions could provide supplementary material for faculty reflection during meta-debriefing activities [30, 31]. In this application, the AI is analysing the debriefing conversation rather than the scenario itself. Meta-debriefing, or “debriefing the debrief”, is increasingly recognised as an importance mechanism for supporting the development of debriefing expertise through structured reflective dialogue among facilitators [32, 33]. AI-supported analytics may therefore offer new opportunities to augment these faculty development processes by providing additional data points for reflection on debriefing practice [34].

**Table:** Case Vignette 2: An example of how facilitators of an MDT focused simulation can use specific LLM outputs to aide a debrief

Case Vignette 2: LLM-assisted debriefing	
An operating theatre team participates in a simulated scenario focused on teamwork during an anaesthetic emergency. The scenario is designed according to crisis resource management (CRM) principles. Following the scenario an AI system analyses the scenario transcript and identifies examples of effective and suboptimal application of CRM principles. It generates a structured summary aligned to the scenario ILOs, along with suggested debriefing topics and open-ended questions. The facilitator uses this output to support the organisation, focus, and structure of the subsequent human-led debriefing discussion.	

##### Limitations

Several limitations are evident. First, transcript-driven or dashboard-based representations of performance may omit contextual nuance, gestural communication, or tacit knowledge, resulting in partial, or occasionally misaligned, outputs. Second, input data quality can be affected by external factors, such as noise interference and technical infrastructure. Third, although intended to reduce facilitator cognitive load, the AI tools may introduce new demands related to transcription workflows, prompt design, input of human judgement of learner performance, software navigation, and critical appraisal of AI-generated content [23]. Fourth, output quality is highly sensitive to prompt quality and framework alignment. Constraining content generation through explicit educational frameworks improves interpretability and reduces output variability [24]. Finally, transcript handling and data storage raise issues of privacy and cybersecurity, while the risk of fabrication, bias, or overgeneralisation necessitates explicit human oversight and appropriate AI literacy among those using the tools [16, 28, 29, 35].

#### 3) Conversational chatbot debriefers

##### Definition and mechanism

Conversational chatbot debriefers are learner-facing AI agents designed to engage directly with learners following a simulation, prompting reflective dialogue in a manner analogous to a human facilitator. These systems typically rely on LLMs combined with structured prompts, scripts, or embedded educational frameworks. Unlike LLM-assisted debriefing, in which AI outputs are physically used by the facilitator or learner, the defining feature of this mode is that the AI assumes the immediate conversational role in the debrief, interacting autonomously with learners during self-led debriefings, generating questions, feedback, and reflective prompts in real time.

##### Current applications

Several studies explored the use of general-purpose LLMs to simulate patients and engage in reflective feedback. Studies utilising LLM powered simulated patients have shown feasibility and reported improvements in communication skills and development of clinical reasoning, while identifying constraints such has character constraints and prompt dependency [36–38]. More purpose-built systems have also emerged. Barker et al. [39] developed the Authentic Teaching and Learning Application Simulation (ATLAS), enabling learners to access simulations asynchronously and revisit prior interactions to build longitudinal continuity with the virtual patient. Another study, implementing an AI-facilitated debriefing agent which uses organised scripted prompts (via Relativ.ai.Inc) to lead students through practice reflection, highlighted the importance of both debrief structure and time spent engaged in the debrief as important factors in shaping outcomes [40]. Others have provided more critical insight into chatbot-led debriefing, observing differences in cognitive load between human- and chatbot-led debriefs and noting tendencies toward overly positive or even sycophantic responses within AI-generated dialogue [41, 42]. While learners often maintained critical self-reflection despite this tendency, the findings highlight potential risks in unmoderated AI-led reflective conversations.

##### Contribution to debriefing

Conversational chatbot debriefers address several practical constraints currently apparent in simulation debriefing practice. They enable repeated, on-demand reflective dialogue without requiring faculty presence, thereby increasing scalability and access. This is particularly valuable for communication skills trainings, where iterative practice and feedback are central. These contributions are echoed in reviews of generative AI in simulation-based education that similarly emphasise scalability and accessibility as key advantages of learner-facing AI systems [27–29]. Additionally, chatbot debriefers may reduce performance anxiety in some learners by providing a more psychologically safe self-governed reflective environment [19]. Furthermore, the ability to revisit simulations asynchronously introduces opportunities for longitudinal reflective development [39], thereby extending the reach of debriefing beyond contexts that are traditionally limited by time and faculty availability.

**Table:** Case vignette 3: An example of how a educators could utilise chatbot simulation and debrief to build challenging communication skills

Case Vignette 3: Conversational chatbot debriefers	
A social work student practices communicating with the families of patients in the final hours of life using a virtual avatar representing a distressed relative. Following the interaction, a chatbot engages the student in a reflective debriefing conversation, guided by a structured rubric developed by the curriculum team. The chatbot provides feedback on communication behaviours, offers suggested phrasing, and prompts further reflection through Socratic questioning. The student repeats multiple scenarios over the week, using the chatbot to support iterative, self-directed learning.	

##### Limitations

Despite these advantages, conversational chatbot debriefers raise significant pedagogical and governance concerns. First, because generative AI models are trained on large and often heterogenous datasets, they are vulnerable to fabrication, overgeneralisation, and bias, particularly when operating without facilitator mediation [29, 35, 43]. They may therefore reproduce patterns and biases embedded within the data, inadvertently reinforcing stereotypes or inequities in training environments [28, 29, 44]. Second, chatbot responses may be overly positive with insufficient levels of critical challenge, potentially impacting the depth of reflective analysis [41, 42]. Third, chatbot systems are currently limited in their capacity to interpret gestural communication, emotional regulation, or relational dynamics, dimensions that are central to high-quality debriefing, particularly in team-based simulations [36].

#### 4) Hybrid and integrated AI debriefing systems.

##### Definition and mechanism

This mode encompasses technologies and terminologies that may be unfamiliar to simulation practitioners. Definitions have been provided in the glossary (Fig. 2). Hybrid and integrated AI debriefing systems refer to purpose-built platforms that combine multiple AI components, such as deterministic rule-based logic, machine learning classifiers, LLMs, multimodal analytics, and structured evaluation frameworks, into a unified debriefing architecture. Unlike metric-based tutors, which rely primarily on telemetry, or conversational chatbot systems, which rely primarily on LLM dialogue, these systems integrate multiple analytic layers to produce structured, often auditable, feedback outputs. Architecturally, such systems may combine event detection rules -augmented generation (RAG) systems, and LLM-based natural language generation. The defining feature of this mode is deliberate systems integration rather than reliance on a single AI component.

##### Current applications

LogiDebrief is one such example of a hybrid system combining logic-enhanced reasoning with LLM generation to produce structured, auditable debriefing reporting for 911 emergency call analysis [45]. Although developed for clinical practice rather than simulation contexts, its architectural principles, particularly the integration of rule-based reasoning and LLM synthesis, appear transferable for debriefing algorithmic simulations such as cardiac arrest. Salvetti et al. [46] described the development of embodied conversational agents (ECAs) that integrate layered natural language processing with GPT-based models. Unlike purely text-based chatbots, ECAs incorporate elements of non-verbal behaviour analysis and can provide both in-scenario guidance and correction, and post-scenario feedback. Similarly, MedSimAI is an integrated platform combining LLM-driven patient simulation, structured feedback mapped to validated communication evaluation tools, and longitudinal self-regulated learning interfaces [47]. MedSimAI separates interaction from evaluation, embedding performance tracking and reflective journaling into a broader learning ecosystem.

##### Contribution to debriefing

Hybrid and integrated AI systems offer several advantages over single-component approaches. First, combining deterministic logic with generative models may enhance reliability, output traceability, and alignment with competency frameworks. Second, multimodal analytics may capture behavioural and contextual data not available through transcripts alone, potentially enriching reflective discussion. Third, integrated platforms enable longitudinal tracking of learner performance, supporting developmental feedback beyond a single simulation event. In this respect, hybrid systems may move AI-driven debriefing beyond augmentation of discrete tasks towards the redesign of reflective architectures. Such integration may support coherence, standardisation, and scalability when implemented within clear governance frameworks [28, 29]. Beyond educational contributions, such analytic capabilities may also have relevance for translational simulation. Translational simulation describes using simulation activities to diagnose and improve healthcare systems, processes, and patient outcomes rather than solely developing individual learner competence [48, 49]. In such contexts, debriefing conversations frequently centre on system behaviours, latent safety threats, and organisational processes. AI-enabled analytics capable of synthesising complex multimodal simulation data may therefore support translational simulation debriefings by identifying patterns in team interaction, workflow performance, or environmental design that may otherwise be difficult to capture.

**Table:** Case vignette 4: An example showcasing how a multidisciplinary team might use hybrid and integrated AI systems to formulate reports from their translational simulation activities

Case Vignette 4: Hybrid and integrated AI debriefing systems	
A multidisciplinary healthcare team participates in an in-situ simulated scenario designed to explore medication safety within a hospital ward. The simulation captures audio, workflow processes, and electronic prescribing interactions. Following the scenario, an integrated AI system analyses these multimodal data to identify patterns in team communication, prescribing behaviours, and system interactions. It generates a structured debriefing report highlighting latent safety threats, including a prescribing workflow vulnerability linked to the electronic health record. During the debriefing, facilitators feed these findings back to the scenario participants, asking for their insights into the reported problems and potential solutions that they would suggest. The debriefing team use this feedback to formulate their simulation report which is fed back to the ward management staff via the appropriate governance channels.	

##### Limitations

Despite their conceptual sophistication, hybrid systems introduce substantial practical and governance challenges. Their technical complexity requires significant expertise, financial investment, and ongoing maintenance. Validation of integrated analytics pipelines remains limited, and few systems have undergone large-scale, independent evaluation. Moreover, while architectural grounding, such as RAG or rule-based constraints, may mitigate hallucination risk, it does not eliminate issues related to bias, data privacy, or interpretive error. As systems become more embedded in assessment and feedback processes, transparency of algorithms, validation of scoring mechanisms, and alignment with curricular competency frameworks become increasingly critical. Finally, greater integration does not remove the need for human oversight. Even highly engineered systems depend on facilitators and programme leaders to interpret outputs, contextualise feedback, and safeguard learner psychological safety. At present, hybrid and integrated AI debriefing systems remain in early developmental stages. Their promise lies in architectural robustness and longitudinal capability, but their implementation demands deliberate governance, validation, and educational alignment Table 1.

#### Practical applications

The integration of AI into debriefing remains in an exploratory phase. As Cheng and McGregor [16] emphasise, responsible adoption of AI in healthcare simulation requires attention to foundational elements including ethics, governance, cybersecurity, and AI literacy. These considerations are particularly pertinent within debriefing contexts, where learner vulnerability, psychological safety, and reflective dialogue are central to the educational process. As with other simulation technologies, the value of AI lies not in the technology itself, but in how intentionally it is aligned with educational objectives, learner needs, and contextual constraints. The following considerations offer practical guidance for simulation practitioners exploring the integration of AI-driven debriefing into their practice.

##### Develop AI-literacy amongst faculty

A foundational requirement for responsible implementation of AI is developing AI literacy among simulation practitioners [16]. AI literacy refers to the competencies required to understand, critically evaluate, and appropriately use AI technologies in an ethical and informed manner [50]. For simulation practitioners, this includes familiarity with the capabilities and limitations of generative AI systems, an understanding of how AI-generated outputs are produced, and the ability to critically appraise those outputs within educational contexts [16, 23].

One practical component of developing AI literacy involves understanding how prompts shape the behaviour of generative AI systems (see Supplement 1 for prompt examples).

##### Align AI modalities with educational aims

**Fig. 1:**
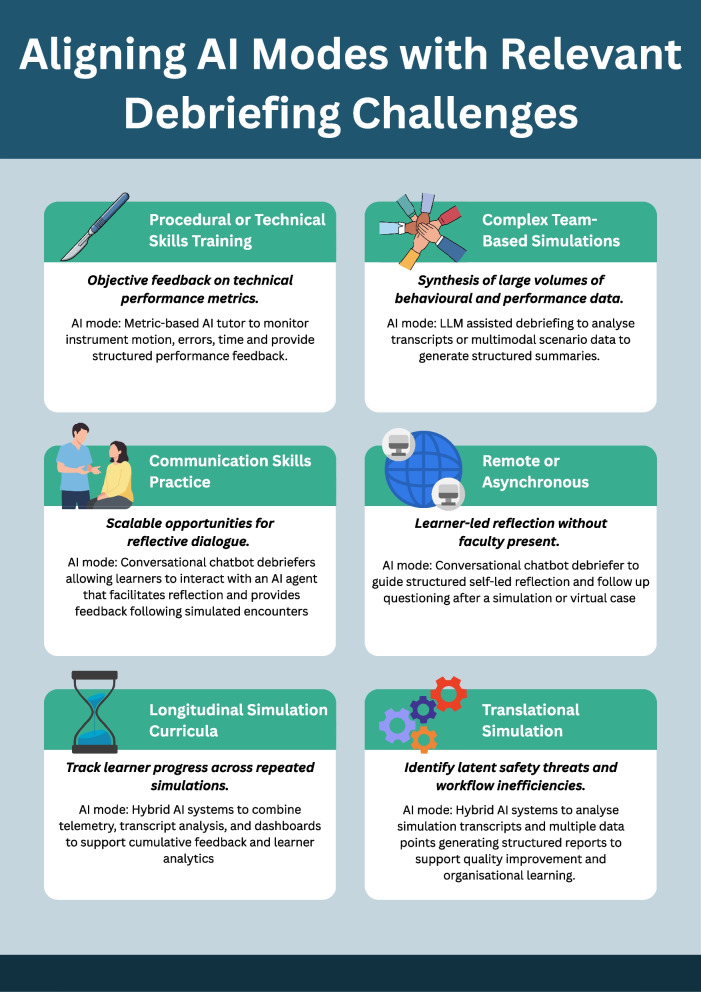
Aligning AI modes with relevant debriefing challenges

**Fig. 2:**
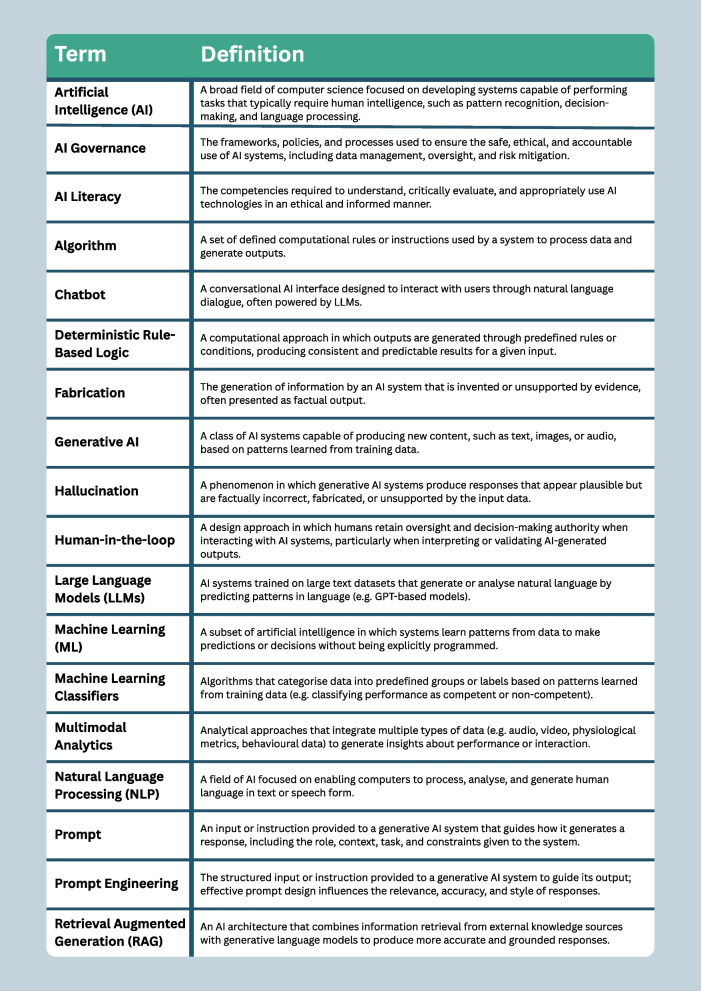
Glossary

Simulation practitioners should carefully consider how different AI approaches align with the educational or organisational aims of their simulation activities (Fig. 1). As described in this article, distinct modes of AI-driven debriefing appear suited to different contexts. Aligning AI modalities with clearly defined learning or organisational objectives therefore represents an important first step in determining how these technologies may enhance existing debriefing practice (Fig. 1).

##### Establish governance, data safety and cybersecurity safeguards

The introduction of AI-driven tools into simulation programmes should be accompanied by robust governance structures [16]. Many AI-supported approaches rely on the processing of sensitive learner data. When these data are processed through external AI platforms, questions arise regarding data ownership, storage, and potential secondary use. Simulation practitioners must therefore ensure appropriate policies for data collection, storage, access, and deletion are in place. Clear informed consent processes should outline how learner data will be used, how AI-generated feedback will contribute to learning, and what safeguards exist to protect learner privacy.

Additional governance considerations include monitoring for potential algorithmic bias, maintaining transparent audit trails of AI-generated outputs, and establishing clear escalation pathways to human facilitators when AI systems generate uncertain or potentially misleading responses. Because many AI systems operate through cloud-based infrastructure and external computational services, simulation programmes must also address cybersecurity risks, implementing safeguards to prevent unauthorised access, data breaches, or misuse of sensitive data [16, 51].

##### Introduce AI-driven debriefing through pilot projects and iterative evaluation

Given the evolving nature of AI technologies, simulation programmes will benefit from introducing AI-driven debriefing through small-scale pilot initiatives. Such pilots allow simulation practitioners to explore the potential value of AI tools while evaluating their impact on learner experience, facilitator workload, and programme outcomes. Encouragement to experiment may be in tension with safeguards previously mentioned, mirroring anxiety for many grappling with AI integration. Pilot projects allow for experimentation while navigation and management of safeguards are concurrently considered and actioned.

Importantly, implementation should be viewed not as a one-time deployment followed by evaluation, but as a process of refinement. AI systems often require continuous adjustment of prompts, workflows, governance structures, and faculty practices in response to user experience and emerging evidence.

##### Maintain human oversight, pedagogical leadership, and personal fulfilment

Finally, the integration of AI-driven tools should not diminish the central role of human facilitators. Debriefing is fundamentally a relational and pedagogical process that relies on contextual judgement, emotional awareness, and the cultivation of psychological safety [1, 20, 52]. Concerns remain regarding the accuracy and reliability of AI-generated outputs, including incomplete, inaccurate, or fabricated outputs, a phenomenon commonly described as ‘hallucinations’ [16, 53]. Maintaining a ‘human-in-the-loop’ approach ensures that facilitators retain interpretative authority over AI-generated outputs and can critically evaluate AI-generated insights and integrate them appropriately within the broader educational context. AI-driven debriefing should therefore be understood as a means of redistributing analytic labour and extending educational capacity, rather than replacing the expertise of simulation educators.

Perhaps the most critical consideration is philosophical- what impact will the use of AI have on our identity and craft? [54]. Even if AI can replace us—doing our debriefings faster, better, and on demand—do we want it to? Simulation debriefing is a microcosm of a more profound contemporary dilemma: as AI takes over complex, creative tasks, individuals may struggle to find personal fulfilment or a sense of unique contribution in their work. Many of us find satisfaction in the challenge of making the relational, contextual, and pedagogical judgments that shape debriefings. Our conversations with learners are satisfying parts of our job. Do we want to spend our days constructing prompts or designing rubrics for AI feedback tools, or would we rather just have the conversations? The potential of AI-driven debriefing to enhance quality, efficiency, scalability and accuracy is significant. Whether it leads to the thoughtful integration of human expertise and intelligent technologies to support reflective learning, or to the complete substitution of human facilitators, however, remains to be seen.

## Conclusions

AI is beginning to reshape multiple aspects of healthcare simulation, including debriefing. In this article we have described four modes of AI-driven debriefing, metric-based AI tutors, LLM-assisted debriefing tools, conversational chatbot debriefers, and hybrid integrated systems, each offering different ways of supporting reflective learning. While the empirical literature remains early and largely exploratory, these approaches demonstrate how AI may augment aspects of debriefing practice by assisting with performance analysis, structuring reflective dialogue, and expanding opportunities for scalable or asynchronous learning.

## Supplementary Information

Supplementary Material 1.

## Data Availability

No datasets were generated or analysed during the current study.
